# Mid- to Long-term Outcomes Following Primary Augmentation With B-Lite Lightweight Breast Implants: A Prospective Multicenter Postmarketing Clinical Follow-up Study

**DOI:** 10.1093/asj/sjag105

**Published:** 2026-07-28

**Authors:** Matthias Reichenberger, Per Hedén, Guenter Germann

## Abstract

**Background:**

Lightweight breast implants (B-Lite) integrate air-filled microspheres into silicone gel to reduce implant weight by up to 30% while maintaining volume and mechanical properties. Long-term prospective data in primary augmentation remain limited.

**Objectives:**

To evaluate clinical performance, safety, satisfaction, and quality of life (QoL) outcomes following primary augmentation with B-Lite implants.

**Methods:**

This multicenter, prospective, single-arm manufacturer-sponsored postmarketing clinical follow-up (PMCF) study enrolled genetically female patients (18-60 years) undergoing bilateral primary breast augmentation. Implants (round or anatomical; POLYsmoooth, POLYtxt, MESMO, or Microthane; 200-755 cc) were placed through inframammary incisions using a standardized technique. The primary endpoint was ≥1 bra-cup increase at 12 months; secondary endpoints included QoL (Body Esteem, Rosenberg, shortened Tennessee, RAND SF-36), satisfaction, and adverse events (AEs) such as capsular contracture (Baker III-IV), rupture, breast implant–associated anaplastic large cell lymphoma (BIA-ALCL), and reoperation.

**Results:**

In all, 101 patients (mean age 32.7 ± 8.0 years; BMI 20.9 ± 1.8) underwent augmentation. At 12 months, ≥1-cup increase occurred in 92% (95% CI, 84%-97%). No Baker III/IV contracture, rupture, or BIA-ALCL occurred. The most frequent AE was transient sensory change (8.9%); others were infrequent and mainly procedure related. Kaplan–Meier reoperation risk was 6.9% at 1 year and 9.9% at 5 years. Patient satisfaction averaged 98.6% with 0% regret; surgeons were satisfied or very satisfied at 97% to 100%. QoL showed durable gains in Body Esteem–sexual attractiveness and Rosenberg; SF-36 composites remained stable.

**Conclusions:**

Lightweight B-Lite implants showed high effectiveness, very high satisfaction, and a favorable safety profile through 3 to 5 years.

**Level of Evidence: 2:**

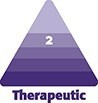

Primary breast augmentation is one of the mostperformed aesthetic procedures worldwide, aimed at enhancing the volume and contour of the female breast. Traditionally, silicone or saline implants have been used, and these have undergone continuous advancements over the past decades to improve aesthetic outcomes as well as safety and biocompatibility.^1-3^ A novel design approach in the development of breast implants is the so-called lightweight implants (B-Lite; POLYTECH, Dieburg, Germany), which are designed to reduce the physical strain on tissue by reducing the weight of the implants. This weight reduction is achieved by integrating air-filled microspheres into the silicone matrix while maintaining the volume and mechanical properties of the implants. The presence of microspheres enables a 25% to 30% reduction in implant weight, compared to other silicone breast implants of the same volume.^4^

The first prototypes were introduced in Israel almost 2 decades ago and have since undergone continuous development, culminating in the acquisition of CE (*conformité européenne*) certification in 2013. The principle of these implants is the use of proven shells (smooth, microtextured, or polyurethane-covered surface), which have been in clinical use for around 20 years with low complication rates, in combination with a completely redesigned filling material.

Although the potential benefits of lightweight implants, such as reduced strain on soft tissue and possibly lower complication rates, appear promising, there is currently limited scientific research systematically analyzing the actual clinical impact of these implants on patients following primary breast augmentation.^4-6^ This prospective study evaluates the effects of B-Lite lightweight implants on postoperative outcomes, complications, and overall patient satisfaction in primary augmentation, representing the first long-term evidence-based evaluation of this new generation of implants.

## METHODS

### Study Design

This is an interim analysis of a multicenter, prospective, single-arm postmarket clinical follow-up (PMCF) study sponsored by G&G Biotechnology Ltd. (Haifa, Israel). The study was conducted to fulfill postmarket surveillance requirements under the European Medical Device Regulation (EU MDR) and corresponding CE-notified body obligations.

The study protocol, consent forms, and relevant documents were approved by independent ethics committees at each site. The study adhered to good clinical practice (GCP) guidelines, the Declaration of Helsinki, and relevant regulatory requirements. This study is registered at clinicaltrials.gov (NCT02777476).

### Study Population

A total of 113 female patients, ages 18 to 60 years, were enrolled across 2 study sites: Ethianum, Heidelberg, Germany, and Akademikliniken, Stockholm, Sweden. Eligible patients were genetically female, seeking primary breast augmentation. Exclusion criteria included active infections, pregnancy or nursing within 6 months before enrollment, significant comorbidities that could pose surgical risks, or a history of psychiatric conditions that could impact informed consent. Demographic and baseline characteristics included patient’s age at the time of surgery, BMI, education level, the number of births, breastfeeding history, smoking status, menopausal status, and reason for surgery.

### Study Intervention

Patients underwent bilateral implantation of the B-Lite lightweight breast implants, round or anatomical shaped with a smooth (POLYsmoooth), microtextured (POLYtxt, MESMO) or polyurethane foam–covered (Microthane) surface, with implant volumes ranging from 200 to 755 cc. Surgical technique, including implant pocket placement (subglandular or submuscular) and choice of implant, was determined by the operating surgeon based on patient anatomy and clinical considerations. Each surgical procedure was conducted in a standardized manner according to the 14-point plan through an inframammary approach, utilizing sharp dissection of the pocket under direct visualization, with meticulous and proactive hemostasis to optimize surgical precision and outcomes.^7^ Standard intraoperative prophylactic antibiotics were administered, and postoperative care followed institutional protocols.

### Study Endpoints

The primary performance endpoint was defined as an increase of 1 or more cup size at the 12-month postoperative follow-up. Secondary endpoints included patient-reported quality of life and satisfaction scores, surgeon-reported satisfaction with the clinical outcome, incidence rates of adverse events, including clinically relevant capsular contracture (Baker III-IV), implant rupture, breast implant–associated anaplastic large-cell lymphoma (BIA-ALCL), and other complications requiring reoperation.

### Assessment Methods

Preoperative and postoperative evaluations included the following:

1. Clinical assessments: Each visit included physical examination, weight, and breast and bra size measurements using a standardized tape measure of breast and chest circumference. Ultrasound (US) examination was performed preoperatively and at 1, 3, and 5 year follow-up.
2. Photographic documentation: Standardized 2-dimensional photographs were taken preoperatively and at each follow-up visit. When available, AP, oblique, and lateral views were captured. For longitudinal comparison, nipple position and inframammary fold levels were assessed with consistent anatomical reference landmarks to evaluate positional stability over time.
3. Patient-reported outcomes: Patient-reported outcomes (PROs) included both validated quality of life (QoL) instruments and a structured study-specific satisfaction questionnaire. All PRO instruments were administered preoperatively (baseline) and at 3, 6, and 12 months postoperatively, and annually thereafter. Validated QoL instruments included the following:
  - Rosenberg Self-Esteem Scale
  - Body Esteem Scale (sexual attractiveness, weight concern, and physical condition domains)
  - Shortened Tennessee Self-Concept Scale
  - RAND SF-36 health survey (8 domains, including physical and mental component summary scores)

These instruments were administered and scored according to their published instructions and validated methodologies. Patient satisfaction was assessed with a structured, prospectively defined study-specific questionnaire. The instrument consisted of 16 items addressing aesthetic outcome, breast appearance (clothed and unclothed), size and proportions, recovery experience, decisional regret, recommendation, and perceived life impact. Responses were recorded using 4-point Likert-type scales (“very satisfied” to “very dissatisfied”) and 3-point agreement scales (“definitely agree” to “disagree”). The questionnaire was not a previously validated instrument but was applied consistently across study sites at all scheduled follow-up visits. All questionnaires were completed by patients during scheduled study visits as part of protocol-defined assessments.

4. Surgeon-reported outcome surveys: Operating surgeons completed standardized satisfaction assessments at surgery and during follow-up visits.

### Study Duration and Follow-Up

Assessments were made postimplantation in 10 days and at 3, 6, 12, 24, and 36 months. At the German sites, patients who provided additional consent were followed up at 48 and 60 months. Patients experiencing adverse events were monitored until resolution or stabilization. Early termination criteria included patient withdrawal, loss to follow-up, or study discontinuation by the sponsor for safety or regulatory reasons.

### Statistical Analysis

Statistical analyses were conducted with JMP Pro Statistical Discovery software (version 17.2.0). Continuous variables were summarized with mean, standard deviation, and range, whereas categorical variables were presented as counts and percentages. The primary endpoint was analyzed with a 2-sided 95% CI based on the Clopper-Pearson method.

To account for changes in sample size with each follow-up visit, all analyses of patient-reported outcomes were conducted on an observed-case basis, with percentages calculated with the number of respondents at each respective time point as the denominator. QoL scales were analyzed with Welch's *t* test comparisons of each timeline to baseline including Bonferroni correction for multiple comparisons adjustments with a CI of 95%. Adverse event rates were assessed descriptively, with reoperation-free survival analyzed by Kaplan-Meier method. No imputations of missing data were performed.

## RESULTS

As of July 2024, 113 patients were enrolled in the study, of whom 101 patients (202 implants) underwent primary breast augmentation with B-Lite implants. Geographical spread was 60% in Germany and 40% in Sweden. The majority of patients completed 3-year follow-up, with approximately 20% completing the 5-year follow-up visit. Table 1 summarizes the demographic and baseline characteristics of the study participants.

**Table 1.** Demographic Characteristics (*n* = 101)

Characteristic	
Age (years), mean ± SD BMI	32.7 ± 8 20.9 ± 1.8
Smoking, %	Yes 22/No78
High school education or higher, %	Yes 98/No 2
Marital status, %	
Married	47.5
Single	42.5
Divorced	4
Separated	5
Widowed	1
Patients with at least 1 live birth, %	Yes 54.55/No 45.5
No. of live births, mean ± S)	1.1 ± 1.2
Breastfeeding in patients with at least 1 live birth, %	Yes 92.7/No 7.3
Menopause, %	Yes 1/No 99
Breast cancer family history, %	Yes 13/No 87

BMI, body mass index; SD, standard deviation.

Patients mean age was 32.7 ± 8 years (range 19.5-56.6) with mean BMI of 20.9 (±1.8). The majority of the patients never smoked (78%). Ninety-eight percent had a high school education or higher. Marital status of the enrolled patients was almost equally divided between single (43%) and married (48%). A total of 54.5% of patients had given birth before surgery, with 92.7% of them having breastfed. Only 1 patient reported being in menopause (Table 1). The primary reason for surgery among 97% of patients was “general breast enlargement,” and ptosis was the primary reason in the remaining 3%.

### Implant and Procedure Characteristics

The majority of implants were placed partially submuscular (dual-plane; 88%). All incisions were inframammary. Anatomical microtextured implants were the most common type (72.3%). Implants ranged in size from 200 to 755 cc with the 255 to 300 cc volume range being the most selected. Mean implanted volume was 299.5 and 300 cc on the left and right sides, respectively. The majority (62%) had a high projection with the remainder divided roughly equally between extra high and moderate projections; 1 patient had low projection implants. Eighty-nine percent of the implants had a POLYtxt surface, 7% MESMO, and Microthane and POLYsmoooth surfaces had 2% each. Table 2 summarizes implant and procedure characteristics.

**Table 2.** Implants and Procedures Characteristics (*n* = 202)

Reason for surgery, %		
General breast enlargement	97	
Ptosis	3	
Incision site		
Inframammary	100	
Incision size (cm), mean ± SD	4.9 ± 0.3	
Device placement		
Subglandular	12	
Submuscular	88	
Volume, (cc), mean ± SD		
Left	299.5 ± 52.5	
Right	300 ± 52.3	
Implant type, %		
Anatomical	72	
Round smooth	2	
Round textured	26	
Surface, %		
MESMO	7	
Microthane	2	
POLYsmoooth	2	
POLYtxt	89	
Shape, %		
Anatomical with low height oval base	2	
Anatomical with round base	70	
Round	28	
Projection, %	Left	Right
Extra high	20	20
High	61	63
Moderate	18	16
Low	1	1

SD, standard deviation.

### Safety Outcomes

Key adverse events (rates per implant) included breast pain (3.46%), seroma (1.98%), hypertrophic scarring (2.97%), implant rotation (2.97%), transient swelling (0.99%), and scar revision (1.48%), with the leading adverse event being sensory changes (mostly transient in the nipples; 8.91%) (Table 3). Most of the key AEs were designated procedure-related, with only 1 case of rotation that was designated as device-related.

**Table 3.** Adverse Events

Key AE categories	Patients with at least 1 event (of 101), *n*	Breasts (of 202), *n*	Rate of events per implant (of 202), %	Reoperation, *n^a^*	Device replacement/removal, *n*
Baker II without surgical intervention	5	7	3.46	0	0
Baker III/IV capsular contracture	0	0	0	0	0
BIA-ALCL	0	0	0	0	0
Breast pain not associated with other complications	6	7	3.46	0	0
Hematoma	1	1	0.49	0	0
Hypertrophic scarring	3	6	2.97	0	0
Infection	1	1	0.49	0	0
Lymphadenopathy	2	2	0.98	0	0
Malposition	1	2	0.98	0	0
Nipple correction	1	1	0.49	1	0
Nipple with unacceptably low sensitivity (high/low)	11	18	8.91	0	0
Position change	1	1	0.49	0	0
Rotation	6	6	2.97	3	2
Rupture	0	0	0	0	0
Seroma	3	4	1.98	2	2
Swelling	2	2	0.99	0	0
Scar revision	**2**	3	1.48	2	0

^a^Two additional reoperations were performed for correction of fat transplantation. AE, adverse event; BIA-ALCL, breast implant–associated anaplastic large cell lymphoma.

No cases of capsular contracture (Baker III/IV) or implant rupture were observed. In addition, no abnormalities or suspected ruptures were detected by ultrasound imaging performed at 12, 36, or 60 months of follow-up. There were no BIA-ALCL cases.

To facilitate interpretation alongside patient-reported outcomes, adverse events (AEs) were aggregated into clinically meaningful postoperative time windows, reflecting the typical evolution of early surgical recovery vs mid- or long-term device-related or surgery-related sequelae. A very early window (0-1.5 months) included hematoma (0.3 month), infections (0.2 month), nipple sensory changes (0-0.6 month); almost all were mild and resolved. An early window (1.5-6 months) included continued sensory changes (0.4-6.3 months); breast pain not linked to other complications (2.3-4.9 months); isolated rotation (3.8 months); and nipple correction (4.1 months). Mid window (6-18 months): hypertrophic scarring (6-8.4 months); seroma (8.2-11.4 months); scar revision (≈12 months); asymmetry (months 12.2); and additional sensory issues (11-13). Late (>18 months): rotation/malposition (25.8-37.9 months); transient swelling (25.7 months); lymphadenopathy (20.9; 36.3 months); Baker grade II capsular changes (10.8, 11.9; 36.5, 52.2 months respectively). The vast majority were mild, frequently judged unrelated to the device, and either resolved or ongoing without sequelae.

The Kaplan-Meier risk for reoperation at 1 year was 6.9% and 9.9% at 5 years, whereas the risk for implant replacement was 4.2% for the entire duration of follow-up. Out of the 101 patients, 3 patients underwent 4 reoperations that required implant replacement of 1 implant (2 cases of seroma and 2 cases of rotation). Four other reoperations in 4 patients (1 implant rotation, 1 nipple correction, and 2 scar revisions) did not require implant replacements.

### Efficacy Outcomes

The primary performance endpoint was defined as change in breast and bra size measurements as measured by the proportion of patients with at least 1 cup size increase at the 1-year follow-up. At least 1 cup size increase at 1 year follow-up was achieved in 92% (CI 95%, 84-97) of cases; at least 5% increase in breast circumference in 97% (CI 95%, 90-99); and at least 10% increase in breast circumference in 93% (CI 95%, 90-99) of cases. In addition, blinded visual examination of 92 patient images at baseline and 1 year by 3 independent surgeons confirmed that 100% of patients had an increase of at least 1 cup size.

### Patient and Surgeon Satisfaction

**Figure 1.**
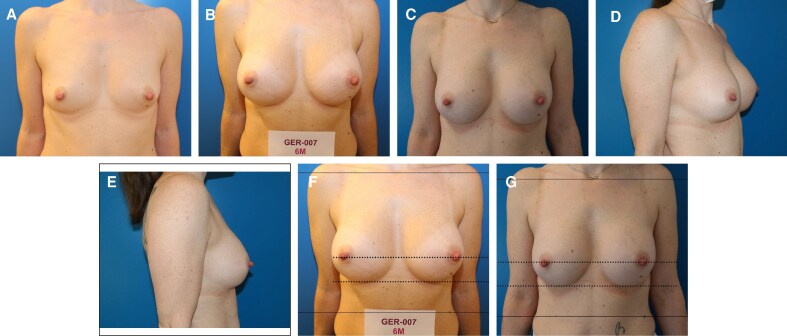
Standardized clinical photographs of a female patient (age 38 years at surgery) following primary breast augmentation with B-Lite implants. (A) Preoperative view (screening), frontal. (B) Postoperative view at 6 months, frontal. (C) Postoperative view at 60 months, frontal. (D) Postoperative view at 60 months, oblique. (E) Postoperative view at 60 months, lateral. (F, G) Comparative frontal views at 6 months and 60 months, respectively, demonstrating sustained implant position and long-term stability (lines indicate maintained implant position over time).

**Figure 2.**
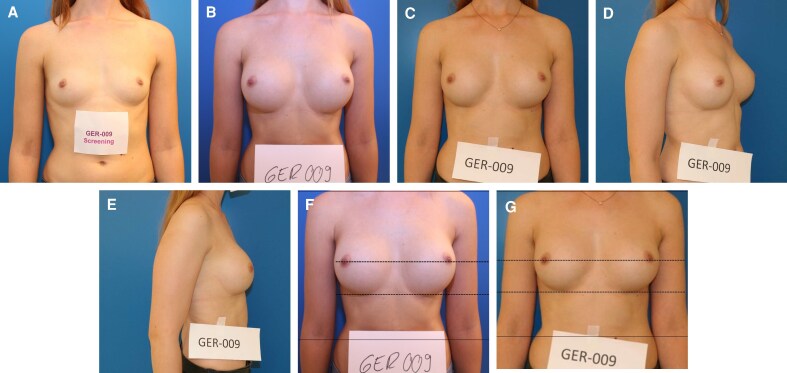
Standardized clinical photographs of a female patient (age 24 years at surgery) following primary breast augmentation with B-Lite implants. (A) Preoperative view (screening), frontal. (B) Postoperative view at 6 months, frontal. (C) Postoperative view at 60 months, frontal. (D) Postoperative view at 60 months, oblique. (E) Postoperative view at 60 months, lateral. (F, G) Comparative frontal views at 6 months and 60 months, respectively, demonstrating sustained implant position and long-term stability (lines indicate maintained implant position over time).

Patients reported high satisfaction regarding aesthetic outcomes, breast appearance (clothed and unclothed), and proportions. The prevalent response across all question categories was “very satisfied” and varied between 71% to 100% throughout all follow-up visits (Supplemental Table 1, available online at https://doi.org/10.1093/asj/sjag105). Overall, positive patient satisfaction averaged 98.6%. Notably, 100% of patients reported no regrets, and 97.8% to 100% felt the surgery positively transformed their lives (Supplemental Table 2, available online at https://doi.org/10.1093/asj/sjag105). The surgeons were very satisfied in 97% and satisfied in 3% of the total 101 cases, with both the implantation procedure and the implant itself on the day of surgery. During the follow-up visits the surgeons were satisfied or very satisfied in 98% to 100% of the cases (Supplemental Table 3, available online at https://doi.org/10.1093/asj/sjag105). Before and after photographs of clinical outcomes are shown in Figures 1 and 2.

### Quality of Life

#### Body Esteem Scale

**Figure 3.**
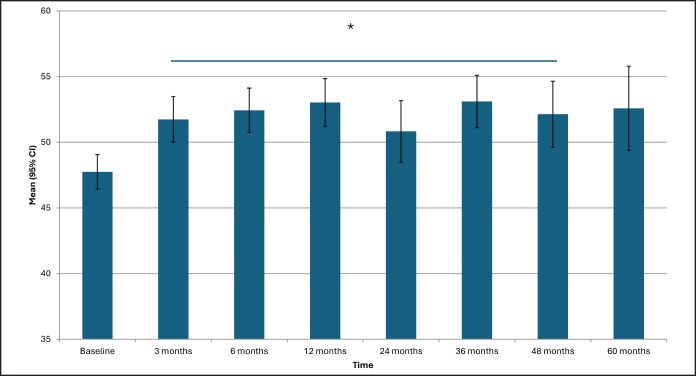
Body Esteem Scale: Sexual attractiveness domain. Bars show mean ± SD; significance (*) assessed by Welch *t* test vs baseline with Bonferroni correction across 7 comparisons.

On the 3 body-esteem subscales, sexual attractiveness showed sustained statistically significant improvements, with Δ = +3.99 (95% CI, +1.83 to +6.15; Bonferroni *P* = .0025) at 3 months, to Δ = +4.39 (95% CI, +1.59 to +7.18; Bonferroni *P*≈.021) at 48 months. No statistically significant changes from baseline were observed for weight or physical condition (Figure 3).

#### Rosenberg Self-Esteem Scale

**Figure 4.**
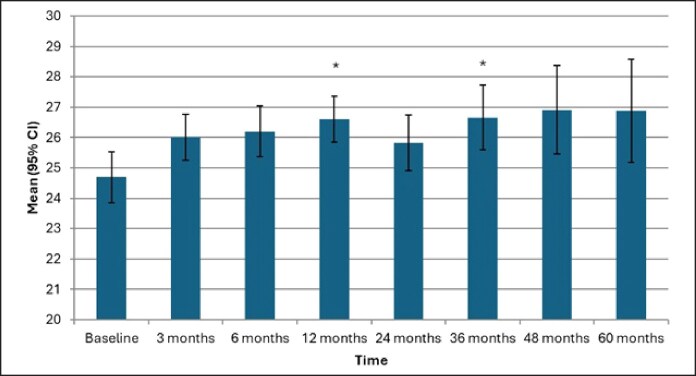
Rosenberg Self-Esteem Scale. Bars show mean ± SD; significance (*) assessed by Welch *t* test vs baseline with Bonferroni correction across 7 comparisons.

A statistically significant improvement was shown at 12 months, Δ = +1.90 (95% CI, +0.78 to +3.03; Bonferroni *P* ≈ .007), and at 36 months, Δ = +1.96 (95% CI, +0.61 to +3.30; Bonferroni *P* ≈ .033). Other time points were positive but not significant after Bonferroni correction (Figure 4).

#### Research and Development Short Form 36 (RAND SF26) Health Survey

Across 3 to 60 months, no Bonferroni-adjusted significant differences from baseline were observed for the physical component summary (PCS) or mental component summary (MCS) (unadjusted MCS at 36 months reached *P* = .019 but was not significant after Bonferroni, *P* = .133.) Of the 8 SF36 domains, physical role at 3 months was lower than baseline (Δ = −14.72; 95% CI, −22.76 to −6.69; Bonferroni *P* < .001). Later time points were not significant after adjustment. Bodily pain at 24 months was lower than baseline (Δ = −8.04; 95% CI, −13.71 to −2.36; Bonferroni *P* = .04). Other time points were not statistically significant different after adjustment. Emotional role at 60 months was higher than baseline (Δ = +7.14; 95% CI, +2.10 to +12.18; Bonferroni *P* = .04). All other domain contrasts did not survive Bonferroni correction (Table 4).

**Table 4.** Bonferroni-adjusted Significant SF-36 Domain Changes Compared With Baseline

SF-36 scale	Time vs baseline	Δ (mean change)	95% CI	*P* (Bonferroni-adj)
Physical role	3 months (*n* = 92)	−14.72	−22.76 to −6.69	*P* < .001
Bodily pain	24 months (*n* = 71)	−8.04	−13.71 to −2.36	*P* = .04
Emotional role	60 months (*n* = 17)	7.14	2.10 to 12.18	*P* = .04

The Δ is follow-up minus baseline (positive = improvement). *P* values were adjusted by Bonferroni for 7 domain and time contrasts tested. All other domain and time contrasts were not significant after adjustment. PCS/MCS showed no Bonferroni-adjusted differences vs baseline. CI, confidence interval; MCS, mental component summary; PCS, physical component summary.

#### Tennessee Self-Concept Scale

Assessment showed no Bonferroni-significant differences vs baseline at any time point. Overall, the QoL battery showed stable global health composites, a transient early physical role decrement (3-month physical role); a mid-term pain decrement (24-month bodily pain); and durable psychological/appearance-related gains (body esteem, sexual attractiveness; Rosenberg at 12 and 36 months). These objective patterns were mirrored by high patient and surgeon satisfaction rates across the 5-year follow-up, with no decisional regret and positive life impact reported.

## DISCUSSION

The clinical impact of implant weight reduction in aesthetic breast augmentation has not been well characterized in prospective long-term studies. This prospective, multicenter investigation therefore provides the first mid- and long-term clinical evaluation of B-Lite lightweight breast implants in primary aesthetic augmentation, with follow-up extending to 5 years.

There are many different implants for aesthetic breast augmentation available, which differ in terms of shape, filling material, and surface characteristics.^2,8-10^ In recent years and decades, the surface of implants has increasingly become the focus of attention, fueled by the debate surrounding BIA-ALCL, which has led to reevaluating the selection of implant surfaces.^11,12^ Terms such as “nano, ” “micro” and “macro” texture are familiar to every breast surgeon today, and the choice of different surfaces has never been greater.^13,14^

The situation is somewhat different when it comes to the filling materials of silicone implants, and quite a few breast surgeons are unaware that there is currently only one company that produces medical silicone suitable and certified for filling of implants. The company NuSil Silicone Technology (Carpinteria, CA, now part of the Avantor group) produces all the long-term implantable medical-grade silicone used as a basic substance by the different implant manufacturers.^15^ The raw material is then further processed by the individual manufacturing companies. The respective base substance can be mixed in various ratios and, depending on the composition of the base substance, softer, firmer, or more dimensionally stable implants are created. A recent development in implant filling technology is the incorporation of microspheres, which significantly reduce the weight of silicone gel–filled implants.

B-Lite implants have been CE approved since 2013 and are now available in more than 60 countries worldwide. This article is based on data from the first prospective, multicenter clinical study performed to evaluate the safety and efficacy of B-Lite implants in the context of primary aesthetic augmentation with 3 to 5 years of follow-up.

The study was challenged by COVID-19 and institutional changes at the Swedish site. Despite that, most patients have completed the 3-year follow-up. The site in Germany has extended the follow-up to 5 years, and 25% of the site's participants have completed 5 years of follow-up, and follow-up of the remaining participants continues.

The demographic profile of our study is in line with data from other similar study designs, with an average age of 32.7 and BMI of 20.9.^16-18^ All patients opted for breast augmentation for aesthetic reasons. No reconstructive or revisional procedures were included in this prospective cohort. Regarding the safety aspects of the implants, there were no cases of implant rupture or breast implant–associated ALCL. Similarly, no cases of grade III/IV capsular fibrosis were observed through 5 years of follow-up in our cohort, in contrast to other long-term reports.^19^ Grade II capsular fibrosis was documented in 5 breasts, which is also considered a low rate. However, the transition from normal and physiologic grade I to grade II capsular fibrosis is a purely subjective matter and is therefore not even mentioned in many other studies.^19^

The low incidence of capsular contracture was an unexpected observation, because the common wisdom is that capsular contracture is correlated with surface characteristics.^11^ The surfaces of the B-Lite implants in our study were equivalent to clinically tested and evaluated shells of POLYTECH implants.^20,21^ Our experience with POLYTECH implants shows a low rate for capsular contracture. However, because we have not followed this systematically, it would be interesting to make a direct comparison with studies in which implants with an identical surface from the same manufacturer were utilized, but with a different implant filling.

Several factors may contribute to the lower incidence of capsular contracture observed in the B-Lite study. First, the reduced weight of B-Lite implants may decrease mechanical stress on the surrounding tissue, leading to lower inflammation and foreign body response. This is supported by biomechanical studies indicating that tissue loading and microtrauma contribute significantly to fibroblast activation and subsequent contracture formation.^22^ Another reason may be the chemical nature of the B-Lite filling, as the gel is less prone to silicone bleeding than conventional fillings.^23^ When examined during consultation, they have a slightly firmer feel, yet contrary to initial expectations most patients reported high levels of positive response (a combination of very satisfied and somewhat satisfied) when asked about natural appearance throughout all follow-ups (94.1%-100% positive response). Finally, all surgeries followed a 14-point plan to minimize perioperative contamination of the implants, which may have contributed to the low capsular contracture rate.^7^

In this study, transient nipple sensitivity changes were reported at a rate of 8.91% per implant or 10.89% per patient. This rate is on the high end of rates typically reported in primary augmentation published data, in which risk estimates for nipple or skin sensation changes vary from as low as 0.0% and 0.4% up to as high as 10.4%.^24-26^ The relatively high rate of sensory changes in our study is plausibly explained by unique active solicitation of this single AE at every visit in our study protocol (forced choice per breast and per nipple), whereas all other AEs were recorded when they occurred or were spontaneously reported. Such methodology increases detection of transient, mild, or expected postoperative sensations that are rarely recorded as AEs in other data sets, and therefore inflates its incidence. Aligning AE collection with standard practice (unsolicited reporting or validated patient-reported outcomes) would likely result in the typical lower rates. Indeed, the rates observed in our study may represent a more accurate estimate of the true incidence, because systematic solicitation likely captures many cases that otherwise go unrecorded. Crucially, these events are well recognized as procedure-related rather than device-specific complications.

No clinically evident rippling, wrinkling, or fold-palpability was observed. Given the single-arm design, this finding cannot be solely ascribed to a device-specific effect and may reflect cohort characteristics, such as low-normal BMI, moderate implant volumes, high-projection selection, or dual-plane placement. These results should therefore be interpreted descriptively.

The reoperation rate of 9.9% is similar or significantly lower compared to other implants.^27,28^ A key factor contributing to this may be the familiar handling characteristics of B-Lite implants, particularly for surgeons experienced with microtextured surfaces, in addition to the physical properties discussed above. Furthermore, these implants do not require any adaptation of the surgical technique compared to other implant systems. For example, because of the reduced pressure on the lower pole the implants do not descend initially like some heavier implants, and therefore placement should be at the location of the permanent desired position (Figures 1, 2).^29^

These findings are consistent with previous reports suggesting that implant weight reduction may contribute to improved postoperative outcomes. Govrin-Yehudain et al. reported favorable outcomes with lightweight implants in aesthetic breast augmentation, and reduced implant weight has also been associated with less postoperative pain and faster recovery.^30,31^ The QoL battery shows stable global health composites, a transient early physical role decrement (3-month physical role); a mid-term pain decrement (24-month bodily pain); and sustained psychological/appearance-related gains (body esteem, sexual attractiveness; Rosenberg at 12 and 36 months). These objective patterns are mirrored by near-ceiling patient and surgeon satisfaction across the 5-year follow-up, with no decisional regret and positive life impact reported.

The adverse-event (AE) time course aligns well with the observed QoL pattern: an early, mostly mild cluster (hematoma/infection, transient sensory change, early pain, normal activity restrictions) maps to the short-term decrease in SF-36 physical role at 3 months, which resolves thereafter; a mid-to-late window that includes seroma requiring interventions, rotation/malposition, and mild capsular changes corresponds to the isolated SF-36 bodily pain dip at 24 months, recognizing that this generic pain domain can reflect non–breast health events as well. Importantly, these largely low-severity, often resolving or unrelated AEs did not blunt the durable psychosocial gains, notably body esteem–sexual attractiveness improvements from 3 to 48 months and Rosenberg self-esteem gains at 12 and 36 months, nor the near-ceiling patient and surgeon satisfaction (98.6% overall patient satisfaction, 0% regret; 97%-100% surgeon satisfaction). Taken together, the AE profile explains transient domain-specific physical effects without undermining the long-term appearance and self-esteem–driven improvements captured by the QoL battery. Late-visit interpretation should consider expected attrition and potential confounding by global health measures.

This investigation has several limitations. First, it is a prospective, single-arm study without a concurrent comparator, so causal inferences vs other implant systems or surgical approaches cannot be drawn and residual confounding variables cannot be excluded. Second, treatment decisions (implant model, projection, pocket, and all cases through an inframammary approach) were surgeon-directed, with most devices being microtextured, high projection, and dual-plane placement. This limits generalizability to other surfaces, shapes, and techniques.

Third, the adverse-event collection mixed routine reporting with active, forced-choice solicitation of nipple sensory changes. Although likely improving detection of transient symptoms, this approach hampers direct rate comparisons with studies employing unsolicited reporting or different patient-reported outcome measures. Future randomized or well-matched comparative studies with fully standardized AE capture would address these constraints.

## CONCLUSIONS

This prospective, multicenter postmarket clinical follow-up study supports the safety and efficacy of B-Lite® lightweight breast implants in primary aesthetic augmentation. No ruptures, BIA-ALCL, or Baker grade III/IV capsular contracture were observed, and patient satisfaction remained high through long-term follow-up. The adverse-event profile was generally favorable and did not appear to undermine sustained improvements in selected psychosocial outcomes. These findings support the use of lightweight implants in primary aesthetic augmentation, while further comparative studies are warranted to evaluate whether the benefits of implant weight reduction extend to reconstructive and revision indications.

## Supplemental Material

This article contains supplemental material located online at https://doi.org/10.1093/asj/sjag105.

## Supplementary Material

sjag105_Supplementary_Data
