# Supplementary material for: Mid- to Long-term Outcomes Following Primary Augmentation With B-Lite Lightweight Breast Implants: A Prospective Multicenter Postmarketing Clinical Follow-up Study
Source: Aesthet Surg J. 2026 Jul 28;46(9):NP88–96. doi: 10.1093/asj/sjag105 (PMC13481097; doi:10.1093/asj/sjag105)
Supplement: Supplementary file 1 [file sjag105_supplementary_data.docx]

**Supplemental Table 1**. Patients Satisfaction With Surgery Results

|  | | Very satisfied | | Somewhat satisfied | | Somewhat dissatisfied | | Very dissatisfied | | Positive response | |
| --- | --- | --- | --- | --- | --- | --- | --- | --- | --- | --- | --- |
|  | Satisfied with | N | % | N | % | N | % | N | % | N | % |
| Month 3 | breast appearance when clothed | 84 | 93.3 | 5 | 5.6 | 0 | 0.0 | 1 | 1.1 | 89 | 98.9 |
|  | recovery process | 78 | 86.7 | 10 | 11.1 | 1 | 1.1 | 1 | 1.1% | 88 | 97.8 |
|  | natural appearance of breast | 78 | 87.6 | 8 | 9.0 | 1 | 1.1 | 2 | 2.2 | 86 | 96.6 |
|  | breast appearance without cloths | 73 | 81.1 | 15 | 16.7 | 1 | 1.1 | 1 | 1.1 | 88 | 97.8 |
|  | breast size | 64 | 71.1 | 22 | 24.4 | 3 | 3.3 | 1 | 1.1 | 86 | 95.6 |
|  | proportions of breast size to body | 81 | 90.0 | 7 | 7.8 | 0 | 0.0 | 2 | 2.2 | 88 | 97.8 |
|  | breast augmentation | 79 | 87.8 | 10 | 11.1 | 0 | 0.0 | 1 | 1.1 | 89 | 98.9 |
| Month 6 | breast appearance when clothed | 78 | 90.7 | 7 | 8.1 | 0 | 0.0 | 1 | 1.2 | 85 | 98.8 |
|  | recovery process | 69 | 80.2 | 15 | 17.4 | 2 | 2.3 | 0 | 0.0 | 84 | 97.7 |
|  | natural appearance of breast | 78 | 90.7 | 7 | 8.1 | 1 | 1.2 | 0 | 0.0 | 85 | 98.8 |
|  | breast appearance without cloths | 66 | 76.7 | 18 | 20.9 | 2 | 2.3 | 0 | 0.0 | 84 | 97.7 |
|  | breast size | 60 | 69.8 | 19 | 22.1 | 7 | 8.1 | 0 | 0.0 | 79 | 91.9 |
|  | proportions of breast size to body | 73 | 84.9 | 10 | 11.6 | 2 | 2.3 | 1 | 1.2 | 83 | 96.5 |
|  | breast augmentation | 76 | 88.4 | 10 | 11.6 | 0 | 0.0 | 0 | 0.0 | 86 | 100.0 |
| Month 12 | breast appearance when clothed | 85 | 95.5 | 3 | 3.4 | 1 | 1.1 | 0 | 0.0 | 88 | 98.9 |
|  | recovery process | 68 | 76.4 | 14 | 15.7 | 5 | 5.6 | 2 | 2.2 | 82 | 92.1 |
|  | natural appearance of breast | 81 | 91.0 | 4 | 4.5 | 4 | 4.5 | 0 | 0.0 | 85 | 95.5 |
|  | breast appearance without cloths | 65 | 73.0 | 16 | 18.0 | 7 | 7.9 | 1 | 1.1 | 81 | 91.0 |
|  | breast size | 66 | 74.2 | 18 | 20.2 | 5 | 5.6 | 0 | 0.0 | 84 | 94.4 |
|  | proportions of breast size to body | 77 | 86.5 | 9 | 10.1 | 3 | 3.4 | 0 | 0.0 | 86 | 96.6 |
|  | breast augmentation | 77 | 87.5 | 8 | 9.1 | 2 | 2.3 | 1 | 1.1 | 85 | 96.6 |
| Month 24 | breast appearance when clothed | 63 | 91.3 | 4 | 5.8 | 1 | 1.4 | 1 | 1.4 | 67 | 97.1 |
|  | recovery process | 54 | 78.3 | 12 | 17.4 | 2 | 2.9 | 1 | 1.4 | 66 | 95.7 |
|  | natural appearance of breast | 59 | 86.8 | 5 | 7.4 | 1 | 1.5 | 3 | 4.4 | 64 | 94.1 |
|  | breast appearance without cloths | 50 | 72.5 | 16 | 23.2 | 2 | 2.9 | 1 | 1.4 | 66 | 95.7 |
|  | breast size | 52 | 75.4 | 13 | 18.8 | 3 | 4.3 | 1 | 1.4 | 65 | 94.2 |
|  | proportions of breast size to body | 60 | 87.0 | 5 | 7.2 | 3 | 4.3 | 1 | 1.4 | 65 | 94.2 |
|  | breast augmentation | 57 | 82.6 | 11 | 15.9 | 0 | 0.0 | 1 | 1.4 | 68 | 98.6 |
| Month 36 | breast appearance when clothed | 43 | 79.6 | 11 | 20.4 | 0 | 0.0 | 0 | 0.0 | 54 | 100.0 |
|  | recovery process | 41 | 75.9 | 11 | 20.4 | 2 | 3.7 | 0 | 0.0 | 52 | 96.3 |
|  | natural appearance of breast | 39 | 76.5 | 9 | 17.6 | 2 | 3.9 | 1 | 2.0 | 48 | 94.1 |
|  | breast appearance without cloths | 36 | 66.7 | 14 | 25.9 | 4 | 7.4 | 0 | 0.0 | 50 | 92.6 |
|  | breast size | 37 | 68.5 | 12 | 22.2 | 4 | 7.4 | 1 | 1.9 | 49 | 90.7 |
|  | proportions of breast size to body | 41 | 75.9 | 10 | 18.5 | 2 | 3.7 | 1 | 1.9 | 51 | 94.4 |
|  | breast augmentation | 43 | 81.1 | 8 | 15.1 | 2 | 3.8 | 0 | 0.0 | 51 | 96.2 |
| Month 48 | breast appearance when clothed | 18 | 85.7 | 3 | 14.3 | 0 | 0.0 | 0 | 0.0 | 21 | 100.0 |
|  | recovery process | 19 | 90.5 | 2 | 9.5 | 0 | 0.0 | 0 | 0.0 | 21 | 100.0 |
|  | natural appearance of breast | 21 | 100.0 | 0 | 0.0 | 0 | 0.0 | 0 | 0.0 | 21 | 100.0 |
|  | breast appearance without cloths | 16 | 76.2 | 3 | 14.3 | 2 | 9.5 | 0 | 0.0 | 19 | 90.5 |
|  | breast size | 15 | 71.4 | 3 | 14.3 | 3 | 14.3 | 0 | 0.0 | 18 | 85.7 |
|  | proportions of breast size to body | 17 | 81.0 | 2 | 9.5 | 1 | 4.8 | 1 | 4.8 | 19 | 90.5 |
|  | breast augmentation | 19 | 90.5 | 2 | 9.5 | 0 | 0.0 | 0 | 0.0 | 21 | 100.0 |
| Month 60 | breast appearance when clothed | 13 | 92.9 | 0 | 0.0 | 1 | 7.1 | 0 | 0.0 | 13 | 92.9 |
|  | recovery process | 12 | 85.7 | 1 | 7.1 | 1 | 7.1 | 0 | 0.0 | 13 | 92.9 |
|  | natural appearance of breast | 12 | 85.7 | 2 | 14.3 | 0 | 0.0 | 0 | 0.0 | 14 | 100.0 |
|  | breast appearance without cloths | 10 | 71.4 | 4 | 28.6 | 0 | 0.0 | 0 | 0.0 | 14 | 100.0 |
|  | breast size | 10 | 71.4 | 3 | 21.4 | 1 | 7.1 | 0 | 0.0 | 13 | 92.9 |
|  | proportions of breast size to body | 11 | 78.6 | 2 | 14.3 | 0 | 0.0 | 1 | 7.1 | 13 | 92.9 |
|  | breast augmentation | 13 | 92.9 | 1 | 7.1 | 0 | 0.0 | 0 | 0.0 | 14 | 100.0 |

**Supplemental Table 2**. Patients Satisfaction With Surgery Results

|  | | Definitely agree | | Somewhat agree | | | Disagree | | Positive response | |
| --- | --- | --- | --- | --- | --- | --- | --- | --- | --- | --- |
| Visit | Question | N | % | | N | % | N | % | N | % |
| Month 3 | Overall surgery was a good experience | 82 | 91.1 | | 8 | 8.9 | 0 | 0.0 | 90 | 100.0 |
|  | No regrets about surgery | 86 | 95.6 | | 3 | 3.3 | 1 | 1.1 | 89 | 98.9 |
|  | Satisfied with the results of surgery | 83 | 92.2 | | 7 | 7.8 | 0 | 0.0 | 90 | 100.0 |
|  | This surgery changed my life for the better | 72 | 80.0 | | 17 | 18.9 | 1 | 1.1 | 89 | 98.9 |
|  | My breasts are the perfect size for me | 67 | 74.4 | | 22 | 24.4 | 1 | 1.1 | 89 | 98.9 |
|  | I would do the surgery again | 75 | 83.3 | | 13 | 14.4 | 2 | 2.2 | 88 | 97.8 |
|  | I feel more attractive than before the surgery | 80 | 88.9 | | 9 | 10.0 | 1 | 1.1 | 89 | 98.9 |
|  | I recommend this surgery to my friends | 71 | 78.9 | | 18 | 20.0 | 1 | 1.1 | 89 | 98.9 |
| Month 6 | Overall surgery was a good experience | 83 | 96.5 | | 3 | 3.5 | 0 | 0.0 | 86 | 100.0 |
|  | No regrets about surgery | 77 | 89.5 | | 8 | 9.3 | 1 | 1.2 | 85 | 98.8 |
|  | Satisfied with the results of surgery | 77 | 89.5 | | 9 | 10.5 | 0 | 0.0 | 86 | 100.0 |
|  | This surgery changed my life for the better | 70 | 81.4 | | 14 | 16.3 | 2 | 2.3 | 84 | 97.7 |
|  | My breasts are the perfect size for me | 62 | 72.1 | | 24 | 27.9 | 0 | 0.0 | 86 | 100.0 |
|  | I would do the surgery again | 76 | 88.4 | | 8 | 9.3 | 2 | 2.3 | 84 | 97.7 |
|  | I feel more attractive than before the surgery | 78 | 90.7 | | 8 | 9.3 | 0 | 0.0 | 86 | 100.0 |
|  | I recommend this surgery to my friends | 68 | 79.1 | | 15 | 17.4 | 3 | 3.5 | 83 | 96.5 |
| Month 12 | Overall surgery was a good experience | 84 | 94.4 | | 4 | 4.5 | 1 | 1.1 | 88 | 98.9 |
|  | No regrets about surgery | 81 | 91.0 | | 6 | 6.7 | 2 | 2.2 | 87 | 97.8 |
|  | Satisfied with the results of surgery | 78 | 87.6 | | 10 | 11.2 | 1 | 1.1 | 88 | 98.9 |
|  | This surgery changed my life for the better | 66 | 74.2 | | 21 | 23.6 | 2 | 2.2 | 87 | 97.8 |
|  | My breasts are the perfect size for me | 66 | 74.2 | | 20 | 22.5 | 3 | 3.4 | 86 | 96.6 |
|  | I would do the surgery again | 83 | 93.3 | | 4 | 4.5 | 2 | 2.2 | 87 | 97.8 |
|  | I feel more attractive than before the surgery | 80 | 89.9% | | 8 | 9.0 | 1 | 1.1 | 88 | 98.9 |
|  | I recommend this surgery to my friends | 71 | 79.8 | | 15 | 16.9 | 3 | 3.4 | 86 | 96.6 |
| Month 24 | Overall surgery was a good experience | 67 | 97.1 | | 2 | 2.9 | 0 | 0.0 | 69 | 100.0 |
|  | No regrets about surgery | 64 | 92.8 | | 5 | 7.2 | 0 | 0.0 | 69 | 100.0 |
|  | Satisfied with the results of surgery | 59 | 85.5 | | 10 | 14.5 | 0 | 0.0 | 69 | 100.0 |
|  | This surgery changed my life for the better | 53 | 76.8 | | 15 | 21.7 | 1 | 1.4 | 68 | 98.6 |
|  | My breasts are the perfect size for me | 53 | 76.8 | | 15 | 21.7 | 1 | 1.4 | 68 | 98.6 |
|  | I would do the surgery again | 64 | 92.8 | | 4 | 5.8 | 1 | 1.4 | 68 | 98.6 |
|  | I feel more attractive than before the surgery | 65 | 94.2 | | 4 | 5.8 | 0 | 0.0 | 69 | 100.0 |
|  | I recommend this surgery to my friends | 54 | 78.3 | | 15 | 21.7 | 0 | 0.0 | 69 | 100.0 |
| Month 36 | Overall surgery was a good experience | 52 | 96.3 | | 2 | 3.7 | 0 | 0.0 | 54 | 100.0 |
|  | No regrets about surgery | 51 | 94.4 | | 3 | 5.6 | 0 | 0.0 | 54 | 100.0 |
|  | Satisfied with the results of surgery | 46 | 85.2 | | 7 | 13.0 | 1 | 1.9 | 53 | 98.1 |
|  | This surgery changed my life for the better | 43 | 79.6 | | 11 | 20.4 | 0 | 0.0 | 54 | 100.0 |
|  | My breasts are the perfect size for me | 35 | 66.0 | | 16 | 30.2 | 2 | 3.8 | 51 | 96.2 |
|  | I would do the surgery again | 50 | 92.6 | | 3 | 5.6 | 1 | 1.9 | 53 | 98.1 |
|  | I feel more attractive than before the surgery | 48 | 88.9 | | 6 | 11.1 | 0 | 0.0 | 54 | 100.0 |
|  | I recommend this surgery to my friends | 39 | 72.2 | | 13 | 24.1 | 2 | 3.7 | 52 | 96.3 |
| Month 48 | Overall surgery was a good experience | 21 | 100.0 | | 0 | 0.0 | 0 | 0.0 | 21 | 100.0 |
|  | No regrets about surgery | 19 | 90.5 | | 2 | 9.5 | 0 | 0.0 | 21 | 100.0 |
|  | Satisfied with the results of surgery | 19 | 90.5 | | 2 | 9.5 | 0 | 0.0 | 21 | 100.0 |
|  | This surgery changed my life for the better | 19 | 90.5 | | 2 | 9.5 | 0 | 0.0 | 21 | 100.0 |
|  | My breasts are the perfect size for me | 17 | 81.0 | | 3 | 14.3 | 1 | 4.8 | 20 | 95.2 |
|  | I would do the surgery again | 19 | 95.0 | | 1 | 5.0 | 0 | 0.0 | 20 | 100.0 |
|  | I feel more attractive than before the surgery | 20 | 95.2 | | 1 | 4.8 | 0 | 0.0 | 21 | 100.0 |
|  | I recommend this surgery to my friends | 17 | 81.0 | | 4 | 19.0 | 0 | 0.0 | 21 | 100.0 |
| Month 60 | Overall surgery was a good experience | 14 | 100.0 | | 0 | 0.0 | 0 | 0.0 | 14 | 100.0 |
|  | No regrets about surgery | 14 | 100.0 | | 0 | 0.0 | 0 | 0.0 | 14 | 100.0 |
|  | Satisfied with the results of surgery | 12 | 85.7 | | 2 | 14.3 | 0 | 0.0 | 14 | 100.0 |
|  | This surgery changed my life for the better | 14 | 100.0 | | 0 | 0.0 | 0 | 0.0 | 14 | 100.0 |
|  | My breasts are the perfect size for me | 11 | 78.6 | | 2 | 14.3 | 1 | 7.1 | 13 | 92.9 |
|  | I would do the surgery again | 14 | 100.0 | | 0 | 0.0 | 0 | 0.0 | 14 | 100.0 |
|  | I feel more attractive than before the surgery | 14 | 100.0 | | 0 | 0.0 | 0 | 0.0 | 14 | 100.0 |
|  | I recommend this surgery to my friends | 12 | 85.7 | | 2 | 14.3 | 0 | 0.0 | 14 | 100.0 |

**Supplemental Table 3**. Surgeon Satisfaction With Implants And Procedures

|  | Very satisfied | Satisfied | Dissatisfied | Very Dissatisfied |
| --- | --- | --- | --- | --- |
| Surgery day  Procedure  Implants | 98 (97%)  98 (97%) | 3 (3%)  3 (3%) | -  - | -  - |
| Day 10 | 86 (88%) | 10(10%) | 2(2%) | 0 |
| Month 3 | 85 (93%) | 6 (7%) | 0 | 0 |
| Month 6 | 73 (80%) | 17 (19%) | 1 (1%) | 0 |
| Month 12 | 77 (86%) | 11 (12%) | 1 (1%) | 1 (1%) |
| Month 24 | 61 (79%) | 15 (19%) | 1 (1%) | 0 |
| Month 36 | 37 (66%) | 18 (32%) | 1 (2%) | 0 |
| Month 48 | 16 (76%) | 5 (24%) | 0 | 0 |
| Month 60 | 12 (86%) | 2 (14%) | 0 | 0 |
